# RNA methylation‐related inhibitors: Biological basis and therapeutic potential for cancer therapy

**DOI:** 10.1002/ctm2.1644

**Published:** 2024-04-04

**Authors:** Huanxiang Chen, Hongyang Liu, Chenxing Zhang, Nan Xiao, Yang Li, Xiangzhuan Zhao, Ruike Zhang, Huihui Gu, Qiaozhen Kang, Junhu Wan

**Affiliations:** ^1^ Department of Clinical Laboratory The First Affiliated Hospital of Zhengzhou University Zhengzhou China; ^2^ School of Life Science Zhengzhou University Zhengzhou China; ^3^ Department of Obstetrics and Gynecology The Third Affiliated Hospital of Zhengzhou University Zhengzhou China; ^4^ Academy of Medical Sciences Zhengzhou University Zhengzhou China

**Keywords:** cancer, drug resistance, inhibitor, RNA methylation

## Abstract

**Abstract:**

RNA methylation is widespread in nature. Abnormal expression of proteins associated with RNA methylation is strongly associated with a number of human diseases including cancer. Increasing evidence suggests that targeting RNA methylation holds promise for cancer treatment. This review specifically describes several common RNA modifications, such as the relatively well‐studied N6‐methyladenosine, as well as 5‐methylcytosine and pseudouridine (Ψ). The regulatory factors involved in these modifications and their roles in RNA are also comprehensively discussed. We summarise the diverse regulatory functions of these modifications across different types of RNAs. Furthermore, we elucidate the structural characteristics of these modifications along with the development of specific inhibitors targeting them. Additionally, recent advancements in small molecule inhibitors targeting RNA modifications are presented to underscore their immense potential and clinical significance in enhancing therapeutic efficacy against cancer.

**Key Points:**

- In this paper, several important types of RNA modifications and their related regulatory factors are systematically summarised.
- Several regulatory factors related to RNA modification types were associated with cancer progression, and their relationships with cancer cell migration, invasion, drug resistance and immune environment were summarised.
- In this paper, the inhibitors targeting different regulators that have been proposed in recent studies are summarised in detail, which is of great significance for the development of RNA modification regulators and cancer treatment in the future.

## Graphical abstract

In this review, several common types of RNA modifications are reviewed, and the role of regulatory factors related to these modifications in cancer progression is discussed. Finally, the latest advances in small molecule inhibitors of these regulatory factors are especially reviewed.

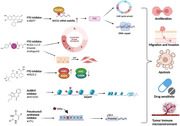

## 1 INTRODUCTION

Cancer is one of the major diseases threatening human health,^1^, ^2^ and the incidence and mortality have increased rapidly in the past 20 years.^3^ There are an average of 12 000 new cancer cases and 10 000 cancer deaths every day, which corresponds to an average of about 7500 cancer deaths every year. In 2020, there will be an estimated 19.3 million new cancer cases (excluding non‐melanoma skin cancers) and nearly 10 million cancer deaths worldwide.^4^ From 2005 to 2020, the total number of deaths due to cancer in China increased by 21.6% to 2 397 772, and the mortality rate and years of life lost increased by 5.0% to 56 598 975.^5^ Despite major advances in the treatment of cancer with various therapies such as surgery, radiotherapy and chemotherapy, the prognosis for most cancer patients is still poor.^2^, ^6^ Therefore, the exploration of new molecular mechanisms affecting cancer is important for establishing new therapeutic options.

Cancer results from the genetic accumulation of different tumour promoters and tumour suppressors. But in recent studies, there is a large body of evidence suggesting that epitope transcriptomics can also influence the cancer process to a large extent. Especially after the emergence of molecular biology, the concept of epitope translatome gradually became known, but RNA modification was not called ‘epitope transcriptome’ until 2015.^7^ Later, through RNA sequencing technology, people gradually discovered the effect of RNA methylation modification on cancer.^8^ Most types of RNA modification are methylated. Mammalian RNA methylation is predominantly N6‐methyladenosine (m6A), N1‐methyladenosine (m1A), N6, 2′‐methyladenosine (m6Am), N7‐methylguanine (m7G), pseudouridine (Ψ) and 5‐methylcytosine (m5C). Studies have shown that these types of modifications are present in various types of RNAs, including non‐coding RNAs (ncRNAs), which in turn affect the metabolism and processing of these RNAs. m6A, as a relatively well‐studied modification type, has been found to be closely related to ncRNA homeostasis and is an important regulator of a variety of cancer‐related signalling pathways.^9^ m6A mainly acts by directly altering the RNA modification of metabolic enzymes and transporters or indirectly affecting related metabolic molecules.^10^ For example, long non‐coding RNAs (lncRNAs) are important regulators of processes such as epigenetic modification, transcription and post‐transcriptional translation and have been shown to be one of the main targets of m6A methylation.^11^ RNA modification was originally designed to adjust the structure of ncRNAs. However, with the discovery of more and more post‐transcriptional regulators, they are now considered to be dynamically regulated. Although RNA modifications are not generally considered critical among the causative factors of cancer, aberrant expression of RNA modifications in terms of function and survival, proliferation, self‐renewal and drug resistance are now found to be signs of cancer.^12^, ^13^, ^14^, ^15^

In recent years, although the impact of RNA modifications on human disease progression has received increasing attention. The mechanisms underlying the role of RNA modification and its associated regulators in cancer are unclear. More and more studies have revealed that RNA modification‐related regulators are also improperly expressed in many human cancers, which brings new ideas for the treatment of human cancer. Many studies point to RNA modification‐related activators or inhibitors as offering new hope for cancer therapy. The same enzymes may act as tumour promoters in one cancer type and as tumour inhibitors in another due to their specific functions in different types of cancer. Therefore, there is a great need to design some specific small molecule inhibitors. Pyrazoline and 5‐fluorouracil have previously been shown to act as inhibitors of dyskerin pseudouridine synthetase 1 (DCK1), providing further insight into the impact of Ψ in cancer therapy. Effective drugs against Ψ are still to be developed. At present, there are relatively many m6A demethylase inhibitors developed. For example, R‐2HG, an fat mass and obesity‐associated protein (FTO) inhibitor, has been shown to have antitumour effects against leukaemia and glioma.^16^ However, there are relatively few studies on m5C inhibitors, and most of them target methyltransferase NSUN2, so the subsequent development of m5C inhibitors is also very promising.

In this review, we will introduce several common types of RNA modifications and their associated regulators. We will further discuss the molecular and cell biological roles played by different modifying enzymes at different levels of RNA modification. Finally, we will list some of the specific small molecule inhibitors that have been developed or are beginning to be used.

## 2 THE REGULATORY NETWORKS OF RNA MODIFICATION

### 2.1 m6A

m6A modification accounts for 60% of RNA methylation modifications and can occur on tRNA, mRNA and rRNA in eukaryotic cells.^17^ Such modifications play an important role in RNA processing and post‐processing transport.^18^ Furthermore, m6A is dynamically reversible.^19^, ^20^ It is mainly controlled by the ‘writers’, ‘erasers’ and ‘readers’. ‘Writers’ mainly include methyltransferase complex, METTL16, WTAP, RBM15/15B, ZC3H13 and ZCCHC4.^21^ The methyltransferase complex consists of the SAM‐binding proteins METTL3 and METTL14.^22^, ^23^ METTL14 recognises RNA substrates and METTL3 is able to catalyse m6A modification of substrates by virtue of a methyltransferase structural domain that it carries, so METTL3‒METTL14 combines to form a heterodimer necessary for methylation.^24^, ^25^ However, ‘erasers’ are mainly composed of FTO and ALKBH5, which can dynamically and reversibly remove m6A modifications.^21^, ^26^, ^27^ ‘Readers’ mainly include YTHDF1/2/3, YTHDC1/2, eIF3 and IGF2BP1/2/3. These proteins generate signals to promote their respective functions by recognising and binding to the m6A site.^28^, ^29^ With the thorough investigation of the binding sites and modification effects of these modifying enzymes, the effects of m6A modification mediated by these modifying enzymes on various RNAs are gradually revealed.

### 2.2 m5C

RNA cytosine can be modified by methylation on its fifth carbon atom, known as m5C modification.^30^ It is found in various RNA molecules.^31^, ^32^ The function of m5C modification on RNA is to maintain the stability of RNA and regulate protein synthesis.^31^, ^32^ The tRNA of m5C can regulate translation, and the rRNA of m5C controls ribosome biosynthesis.^32^ For coding RNA, m5C modification can affect its structure, stability and translation process.^32^ The m5C is similarly regulated by the ‘writers’, ‘erasers’ and ‘readers’.^30^ ‘Writers’ mainly include the DNMT2 and NOP2/NSUN families. ‘Erasers’ refer to the TET family and ALKBH1.^33^ In ‘readers’, ALYREF and YBX1 have been identified.^33^, ^34^ It should be noted that current studies on m5C mainly focus on targeting tRNA and rRNA.

### 2.3 m7G

Another methylation modification is termed N7‐methylguanosine (m7G). The m7G modification is involved in regulating biological functions by modulating the metabolism of multiple RNA molecules.^35^, ^36^, ^37^, ^38^ However, the study of m7G‐modifying enzymes is still in its infancy. The most studied m7G methyltransferase in mammals is METTL1/WDR4 and WBSCR22/TRMT112.^39^ METTL1 binds to WDR4 to induce m7G modification of various RNAs.^39^ In addition, RNA guanine‐7 methyltransferase (RNMT) and RNMT cofactor (RAM) are also involved in m7G modification in mammals.^40^ WBSCR22 and TRMT112 mainly mediate the m7G methylation of rRNA.^41^

### 2.4 m1A

The m1A modification is widely present in transcribed and non‐transcribed RNAs as well as in mitochondrial transcripts.^42^, ^43^ In mRNA, it occurs mainly in the 5ʹ‐UTR region, which is abundant in mRNA and influences translation.^44^ The m1A modification is also affected by the ‘writer’ (TRMT10C), ‘erasers’ (ALKBH1, ALKBH3) and ‘readers’ (YTHDF1/2/3).^42^

### 2.5 Pseudouridine

**FIGURE 1:**
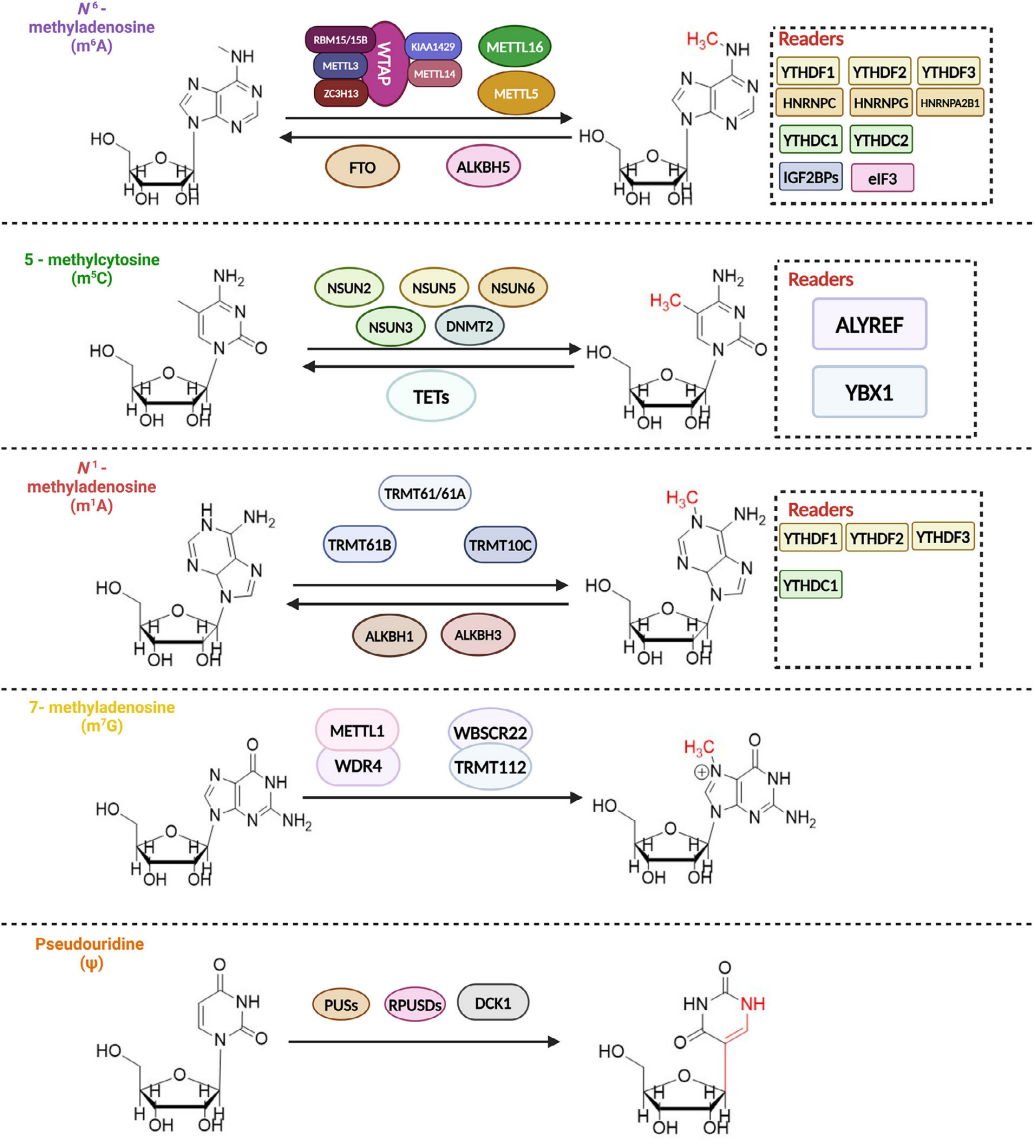
Several common types of RNA methylation and their associated regulators. The methylation process is regulated by methyltransferases. The demethylation process is regulated by demethylases. ‘Reader’ is a methylation recognition protein. In N6‐methyladenosine (m6A) methyltransferase, RBM15/15 b, METTL3, ZC3H13, METTL14, KIAA1429 and WAP play a role in the form of the polymer. In N7‐methylguanosine (m7G) methyltransferases, METTL1 and WDR form aggregates, and TRMT112 and WBSCR22 form aggregates.

Ψ is a natural structural analogue of uracil nucleoside, and ribose is attached to C5 of the pyrimidine ring. This modified form of Ψ was initially found to be present mainly in some ncRNAs, and with the development of detection techniques, it has been found that Ψ can modify almost all RNAs, including mRNAs.^45^ There are six families of Ψ ‘writers’, namely PUS, RSUA, TruA/B/D and RluA.^46^, ^47^ Another ‘writer’ is DCK1, which catalyses Ψ formation using conductive RNA and complementary proteins with modified sequences.^48^, ^49^ The current ‘readers’ and ‘erasers’ of Ψ are not well studied. In eukaryotes, substrates of RNA become pseudouridine mainly in two ways: RNA dependent and RNA independent. Independent RNAs recognise and catalyse substrates directly through PUSs. However, the other requires Box H/ACA RNP catalysis.^50^ Functionally, such modifications can affect aspects of RNA stabilisation and translational control.^51^ Notably, pseudouridine also plays an important role in regulating pathobiological functions in humans^52^, ^53^ (Figure 1).

## 3 THE EMERGING ROLES OF RNA METHYLATION AS CRITICAL REGULATORS OF TUMOURIGENESIS

Current evidence suggests that in malignant tumours, aberrant RNA modification is strongly associated with tumourigenesis or suppression, including proliferation, invasion, metastasis or immune system evasion. Therefore, RNA methylation‐related regulators have become a promising target for cancer therapy. By studying the relevant modifying enzymes of different modification types and exploring their mechanism of action, the corresponding small molecule inhibitors can be designed.

### 3.1 Cell proliferation

#### 3.1.1 m6A

Different m6A methylation‐modifying enzymes were found to have unique pro‐ and oncogenic mechanisms. It is important to begin by describing the promotional role played by some regulatory factors in tumour proliferation. For example, METTL14 can mediate m6A modification of LINC00942 (LNC942) in breast cancer (BRCA), and LNC942 acts as an oncogene that promotes methylation during BRCA occurrence and development. It also regulates the stable expression of its target genes CXCR4 and CYP1B1.^54^ Another well‐studied m6A methyltransferase is METTL3. In nasal cancer, METTL3 promotes the proliferation of nasal cancer cells by regulating the m6A modification of miR‐19a‐3p and further reducing the expression of BMP‐activated hormone membrane‐bound inhibitor homologue (BAMBI).^55^ In addition to the two most common methyltransferases mentioned above, knockdown of METTL16 has been found to reduce the overall level of m6A, which in turn reduces the stability of the cell cycle protein D1 mRNA, leading to reduced levels of the cell cycle protein D1, G1/S arrest and inhibition of proliferation.^56^ In addition to methyltransferases, the role of recognition proteins should not be overlooked. For instance, YTHDF1 identifies and attaches to m6A modification sites in non‐small cell lung carcinoma(NSCLC) and enhances NSCLC cell proliferation by regulating the translational efficiency of CDK2/4 and cyclin D1.^57^ The role of demethylases in promoting cell proliferation is also of interest. For example, expression of ALKBH5 affects the proliferation of glioma stem cells (GSCs).^58^

In addition, in different contexts, these modifying enzymes may also exert an inhibitory effect on cell proliferation. For example, in proliferative vitreoretinopathy, high expression of METTL3 triggers epithelial‐mesenchymal transition (EMT) by affecting transforming growth factor‐β (TGFβ1), which in turn regulates the Wnt/β‐catenin pathway, and impairment of EMT ultimately inhibits cell proliferation.^59^ Another methyltransferase, METTL14, inhibits tumour progression in clear cell renal cell carcinoma (ccRCC) by mediating m6A modification of Phosphatase and tensin homolog (PTEN) mRNA.^60^ Besides methyltransferase, demethylase also plays an crucial role in inhibiting cell proliferation. Upregulation of ALKBH5 has been reported to inhibit cell proliferation by enhancing SOCS3 expression through YTHDF2 recognition.^61^ Furthermore, in diffuse large B cell lymphoma (DLBCL), ALKBH5 promotes TRERNA1 expression by demethylating its potential downstream targets and further inhibits DLBCL proliferation by suppressing p21 expression.^62^ Besides ‘writers’ and ‘erasers’, the role of ‘readers’ cannot be ignored. For example, in hepatocellular carcinoma (HCC), the recognition protein YTHDF2 directly recognises and binds to the m6A modification site of epidermal growth factor receptor (EGFR), destabilising and degrading it, thereby inhibiting cell proliferation and growth.^63^

#### 3.1.2 m5C

Due to the continuous progression of sequencing technology, the effects of m5C regulators on cancer cell proliferation have been further investigated. For example, one study found that NSUN2 was upregulated in gastric cancer (GCs), and by targeting PIK3R1 and PCYT1A, NSUN2 promoted the proliferation of GCs.^64^ Furthermore, NSUN2 was upregulated in both cellular and tissue specimens of uveal melanoma (UM), and β‐catenin (CTNNB1) was a direct target of NSUN2 action. Therefore, an experiment was designed to knockdown NSUN2, and it was found that UM cell migration was inhibited, and cell proliferation was inhibited by cell cycle G1 arrest.^65^ In addition, TRDMT1 is a methyltransferase specific for tRNA, and one study found that knockdown of the TRDMT1 gene resulted in the inhibition of a number of genes involved in cell proliferation.^66^ Similarly, m5C demethylase plays a significant role in specific cancers. For example, ALYREF is highly expressed in bladder cancer. Mechanistically, high ALYREF expression can promote bladder urothelial carcinoma (BLCA) proliferation by regulating PKM2‐mediated glycolysis.^67^

#### 3.1.3 m1A

There are relatively few reports on the effects of m1A‐related modifying enzymes on cell proliferation. For example, m1A‐related enzymes have been found to regulate ErbB and mammalian target of rapamyclin (mTOR) pathways in gastrointestinal cancers; and it has been shown that knockdown of ALKBH3 did inhibit the expression of ErbB2 and AKT1S1. Thus, the proliferation of GI tumours is affected.^68^ In addition, tRNA‐m1A modification has been shown to promote T‐cell amplification through efficient MYC protein synthesis. They found that TRMT61A and TRMT6 are upregulated when T cells leave quiescence, which allows early expression of tRNA in m1A to increase translational efficiency and enable rapid synthesis of MYC. Then, MYC protein can guide naive T cells to activate from a quiescent state and proliferate rapidly.^69^ Another methyltransferase, TRMT10C, has been shown to have an important effect on gynaecologic tumourigenesis. Inhibition of TRMT10C expression inhibits the proliferation of ovarian and cervical cancer cells.^70^

#### 3.1.4 m7G

Similarly, as a form of modification, m7G has been gradually paid attention to, and studies have begun to explore the effects of its modifying enzymes on cell proliferation. For example, it has been shown that WDR4 inhibits the proliferation of HCC cells, and high expression of WDR4 in HCC promotes the binding of EIF2A to CCNB1 mRNA and leads to P53 ubiquitination.^71^ In addition, another METTL1, is also of concern. METTL1 has been found to be a key candidate gene for the overall decrease of m7G in ischaemic mRNA. METTL1 affects puromycin uptake efficiency via m7G tRNA modification, thereby influencing selective oncogene translation and promoting neuroblastoma development.^72^ In addition, the synergistic effect of METTL1 with WDR4 is of interest. For example, in HCC, inhibition of METTL1 or WDR4 expression suppresses HCC proliferation.^35^ Furthermore, METTL1 and WDR4 were able to increase the m7G modification of tRNAs and further promote the translation of oncogenic transcripts associated with the autophagy pathway, which ultimately promoted the proliferation of esophageal squamous cell carcinoma.^73^ Furthermore, in lung cancer, METTL1/WDR4 deletion leads to impaired tRNA m7G modification, which affects lung cancer cell proliferation and tumourigenesis.^36^

#### 3.1.5 Pseudouridine

The related modification enzymes that cause pseudouridine also have opposite effects in different cancer types, which are controlled by different mechanisms. In colorectal cancer, DKC1 is an important regulator affecting colorectal cancer cell proliferation. Proteomics and RNA immunoprecipitation sequence analyses revealed that DKC1 binds to and stabilises the expression of mRNAs for a variety of ribosomal proteins, thereby accelerating the proliferation of cancer cells.^74^ Similarly, PUS7 was also found to promote CRC proliferation by interacting with Sirtuin 1.^75^ In prostate cancer, bioinformatics and expression correlation analyses showed that the lncRNA prostate cancer‐associated transcript 1 also interacts with the DKC1 protein and promotes proliferation and invasion.^76^ Mutations in the DKC1 gene inhibit cell proliferation, leading to X‐linked congenital keratosis pilaris, which remains incurable.^77^

Of course, it is worth mentioning that these modifying enzymes may also play an inhibitory role. Methyltransferase PUS7 is a targeted external transcriptome regulator of glioblastoma growth. PUS7 modifies tRNAs in glioblastoma, reduces TYK2 translation and downregulates proliferation by limiting the interferon‒Stat1 pathway.^78^ In addition, TruB1 regulates the maturation of let‐7 miRNA, a conserved microRNA that is involved in various biochemical processes, including tumour suppression, by mediating post‐transcriptional gene silencing.^79^

### 3.2 Cell migration and invasion

#### 3.2.1 m6A

In addition to attracting attention in cell proliferation, there are also many studies on the effects of m6A modifying enzymes on cell migration and invasion. Consistent with its effect on cell proliferation, m6A modification also has dual effects on cell migration and invasion activities. The first is the inhibitory effect. For example, knockdown of the methyltransferase METTL3 has been observed to promote migration and invasion of human embryonic stem cell (hESC), whereas cell migration and invasion are enhanced by downregulation of METTL3 to promote the development of endometriosis.^80^ Furthermore, METTL14, another methyltransferase, is also an important regulator. A series of experiments showed that overexpression of METTL14 inhibited GC proliferation and invasion.^81^ Also receiving much attention are demethylases. For example, the demethylase ALKBH5 is highly expressed in PC and is dependent on the recognition of YTHDF2 to activate PER1, and the highly expressed PER1 inhibits cell migration and invasion by activating the ATM‒CHK2‒P53/CDC25C axis.^82^ Furthermore, in bladder cancer cells, ALKBH5 knockdown promotes bladder cancer cell migration via CK2‐mediated glycolysis.^83^

Second, the role of promoting cell migration should be explained. For example, METTL3 is able to ultimately promote CRC migration and invasion by inhibiting the expression of the anticancer gene SPRED2.^84^ In addition, it was shown that the expression of METTL3 and c‐Myc could affect LUC migration and invasion through a positive feedback loop.^85^ In addition, it was found that high expression of the m6A demethylase FTO in human BRCA could mediate demethylation degradation of the oncogene BNIP3 and thus promote BRCA cell migration.^86^

#### 3.2.2 m5C

Similar to m6A, m5C modifications affect tumour migration and invasion. NOP2 and NSUN2 were found to promote prostate cancer progression by affecting ubiquitin‐mediated genes associated with the p53 signalling pathway, prostate cancer proteolysis and RNA degradation.^87^ Furthermore, it was found that NSUN2 could regulate the m5C modification of the oncogene FOXC2‐AS1 and make it bind to YBX1 during GC progression.^88^ Notably, NSUN2 deletion in cervical cancer also significantly inhibited cancer cell migration and invasion. Mechanistically, YBX1 recognises NSUN2‐induced m5C methylation of keratin 13 (KRT13) transcripts, enabling its stable expression and ultimately promoting cervical cancer survivability.^89^ Furthermore, in hypopharyngeal squamous cell carcinoma (HPSCC), upregulation of NSUN2 alters the m5C modification of its downstream gene TEAD1, which ultimately promotes HPSCC proliferation and invasion.^90^ More interestingly, there is also a synergistic effect between various modifying enzymes. For example, cross‐regulation between the recognition protein ALYREF and the methyltransferase NSUN2 promotes migration and invasion of BLCA by facilitating splicing and stabilisation of RABL6/TK1 mRNA.^91^

#### 3.2.3 m1A

Compared with the research on the modification content of m6A, there are few studies on the different modification enzymes of m1A and their roles in different types of cancers. However, it has been found that glioma development is closely related to dysregulation of m1A. Inhibition of TRMT6 was found to be involved in glioma survival by regulating cell cycle and cellular pathways such as PI3K‒AKT.^92^ Furthermore, ALKBH3, an m1A demethylase, was confirmed to have a regulatory effect on xenograft growth in vivo. Studies have shown that demethylation of the m1A modification of tRNA is more sensitive to angiopoietin, and the subsequent generation of tRNA‐derived small RNAs around the anticodon enhances ribosome assembly and prevents Cyt c‐induced apoptosis.^93^

#### 3.2.4 m7G

Studies on the relationship between m7G methylation modifications and cancer progression are scarce. One of these studies demonstrated that WDR4 overexpression in HCC promoted cell migration and invasion by affecting the downstream target m7G methylation level, G2/M phase transition, apoptosis and EMT.^94^ It has also been found that METTL1 methyltransferases can mediate m7G methylation in miRNAs, thereby regulating cell migration and invasion.^95^ In addition, METTL1‐mediated m7G modification is regulated by mature let‐7e, and knockdown of it leads to high expression of its target genes HMGA2 and MYC metastasis driver, which inhibits lung cancer cell migration and invasion.^95^, ^96^

#### 3.2.5 Pseudouridine

In the modification of pseudouridine, the effect of DCK1 as a ‘writer’ on cell migration and invasion has also been noted. Although Dyskerin acts more as a tumour suppressor.^97^, ^98^ But other studies have also suggested a carcinogenic effect. For example, in glioma, increased DKC1 expression promotes glioma progression by inducing glioma cell growth and migration.^99^ It was also shown that DKC1 was able to further promote NB cell survival by increasing SNORA50C expression.^100^ In addition, SNORA70E binds DKC1 to regulate Ras‐associated protein 1B (RAP1B) mRNA and increase RAP1B protein level through pseudouridine modification, thereby promoting cancer cell progression in ovarian cancer.^101^

### 3.3 Tumour drug resistance

#### 3.3.1 m6A

m6A modification also has a corresponding effect on drug resistance in cancer cells. For example, METTL3 acts to increase the expression of YAP, which induces drug resistance in NSCLC.^12^ In addition, METTL3 acts to enhance the resistance of BRCA to doxorubicin.^102^ Meanwhile, METTL3 can also inhibit the expression of LATS2 by regulating the lncRNA snhg17 and improve the resistance of lung adenocarcinoma to gefitinib.^103^ Tumour‐associated macrophages in colorectal cancer have also been found to promote oxaliplatin resistance by influencing METTL3‐mediated TRAF5 m6A modification and programmed necrosis.^104^ For another methyltransferase, METTL14, m6A‐modified TRAF1 was found to enhance sunitinib resistance in renal cell carcinoma by regulating apoptosis and angiogenesis.^105^ It has also been found that a methyltransferase, METTL7A, is present in myeloma cancer cells (MM), which promotes the encapsulation of lncRNA into adipocyte exosomes by regulating lncRNA m6A methylation. And this adipocyte‐rich microenvironment exacerbates myeloma drug resistance.^106^ Furthermore, in iodine‐refractory papillary thyroid carcinoma, IGF2BP2 activation leads to resistance to tyrosine kinase inhibitor treatment.^107^ For the demethylase FTO, it was found that upregulation of FTO further enhances ZEB1 expression by decreasing RNA methylation, thereby increasing chemotherapy resistance in tumour cells.^108^ In addition, ALKBH5 has been reported to form an ALKBH5–HOXA10 cycle with its upstream factor HOXA10, which enhances cisplatin (CDDP) resistance in ovarian serous cystadenocarcinoma (OV) by mediating JAK2 m6A demethylation.^109^ Furthermore, m6A modification can also improve resistance to targeted therapies. For example, tyrosine kinase inhibitor (TKI) and mTOR inhibitors are often used in the treatment of ccRCC, but drug resistance is easy to reduce the therapeutic effect. They subsequently demonstrated that suppression of expression of both parental genes by inhibiting methylation of PTEN promoter and GLUT1 resulted in suppression of ccRCC progression and resistance to mTOR inhibitors.^110^ Chemotherapy is an important means of cancer treatment, but drug resistance often occurs in the process of treatment. For example, some studies have found that although CDDP‐based combination chemotherapy has become an important treatment for bladder cancer patients. However, drug resistance often occurs during treatment, leading to treatment failure, which limits its application and effectiveness in bladder cancer.^111^ In addition, gefitinib resistance also occurs during the treatment of lung cancer. Knockdown of virus‐like m6A methyltransferase‐associated protein (KIAA1429) can inhibit the expression of MAP3K2 by recognising m6A methylation and inhibit gefitinib resistance.^112^ Immunotherapy also promotes tumour tolerance as regulatory T cells express the CTLA‐4 immune checkpoint receptor, in which m6A‐modified circQSOX1 promotes PGAM1 expression, which in turn facilitates immune escape from CRC through activation of glycolysis and an anti‐CTLA‐4 therapeutic response, thereby promoting CRC tumourigenesis.^113^

#### 3.3.2 m5C

There are relatively few studies on m5C‐related modifying enzymes in drug resistance. For example, in mouse tumour models, NSUN2 deficiency increases the sensitivity of tumour cells to 5‐Fu and CDDP drugs.^114^ In addition, knockdown of NSUN2 and METTL1 attenuated cellular resistance to CDDP in HeLa cell line.^115^ In oesophageal cancer, NSUN2 enhances chemoresistance to cisplatin in oesophageal squamous carcinoma by regulating lncRNA methylation.^116^ In addition, m5C‐modified mRNAs may also be recognised by YBX‐1 and disrupted by SIAH1, which undergoes ubiquitination at lysine 304, thus impairing the resistance of EOCSCs to CDDP.^117^

#### 3.3.3 m7G

**FIGURE 2:**
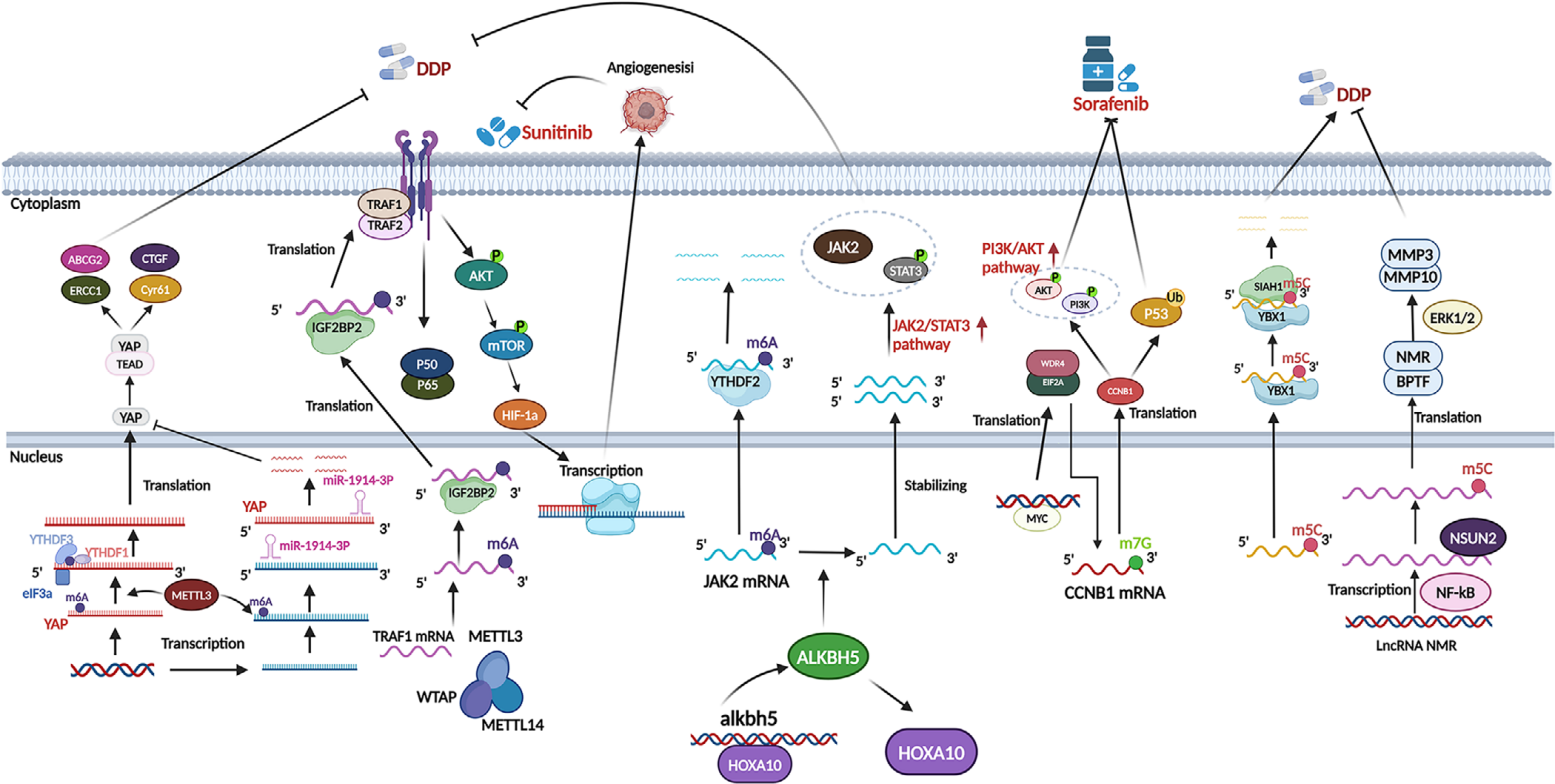
The related regulators of N6‐methyladenosine (m6A), 5‐methylcytosine (m5C) and N7‐methylguanosine (m7G) are involved in the signalling pathways of drug resistance during tumour treatment. The JAK/STAT3 signalling pathway is the downstream pathway of cytokine signalling, which regulates cell development, differentiation, proliferation, apoptosis, etc. It not only participates in the regulation of normal physiological processes, but also plays an important role in the occurrence and development of tumours. PI3K/AKT signalling pathway is also one of the important signal transduction pathways in cells, and its main role is to inhibit cell apoptosis and promote proliferation. PI3K/AKT signalling pathway is abnormally regulated in a variety of malignant tumours, and then promotes tumour cell proliferation and neovascularisation, inhibits cell apoptosis, and is closely related to tumour invasion and metastasis.

This section will enumerate the effects of some modifying enzymes of m7G on cell resistance. For example, overexpression of METTL1 attenuates COAD resistance to CDDP by affecting the P53 pathway.^118^ m7G methyltransferase WDR4 promotes sorafenib resistance in HCC by promoting CCNB1 mRNA methylation, improving its stability and translation.^71^ In addition, upregulation of METTL1 and WDR4 promotes translation of EGFR pathway genes, which triggers levatinib resistance.^119^ In addition, the expression of the complex formed by METTL1 and WDR4 was significantly increased in nasopharyngeal carcinoma (NPC). By upregulating METTL1, it was able to promote EMT in NPC, thereby increasing NPC resistance to CDDP and docetaxel^120^ (Figure 2).

### 3.4 Tumour immunotherapy

#### 3.4.1 m6A

During tumour development, tumour cells monitor immune evasion through the expression of immune checkpoint inhibitors, which are the main mechanism for suppressing the immune response.^121^ The role and mechanisms of m6A‐associated modifying enzymes in tumour immunity are currently the subject of relatively more studies. For example, methyltransferase METTL3‐mediated activation of PD‐L1 mRNA is dependent on the recognition of IGF2BP3. Additionally, research has shown that inhibiting METTL3 or IGF2BP3 can improve antitumour immunity by affecting T‐cell activation, clearance and infiltration through PD‐L1.^122^ Previous studies have shown a positive correlation between the expression level of METTL3 and effector molecules in tumour‐infiltrating NK cells.^123^ For METTL14, macrophage‐specific elimination of METTL14 was found to impair tumour clearance by CD8+ T cells due to dysfunction‐driven CD8+ T‐cell differentiation.^124^ In addition, ALKBH5 suppressed the immune function of antitumour T cells in intrahepatic cholangiocarcinoma (ICC) by upregulating PD‐L1 expression.^125^ Interestingly, ALKBH5 can regulate the tumour microenvironment in response to tumour immunotherapy by inhibiting immune cell aggregation.^115^ In addition, another demethylating enzyme, FTO, counteracts the response to PD‐1 blockers in melanoma patients.^126^

#### 3.4.2 m5C

Among the m5C‐modifying enzymes, NSUN3 and NSUN4 are associated with six major immune cell infiltrates, with NSUN3 and NSUN4 being strongly associated with CD8+ T cells and neutrophils, respectively.^127^ Furthermore, NSUN2 was negatively correlated with the infiltration of immune cells in the nasopharyngeal carcinoma tumour microenvironment, suggesting that high expression of NSUN2 may reduce the sensitivity to immunotherapy.^128^ Furthermore, tissue microarray showed that high expression of DNMT1 in tumour tissues was positively correlated with many immune cells.^129^

#### 3.4.3 m7G

**FIGURE 3:**
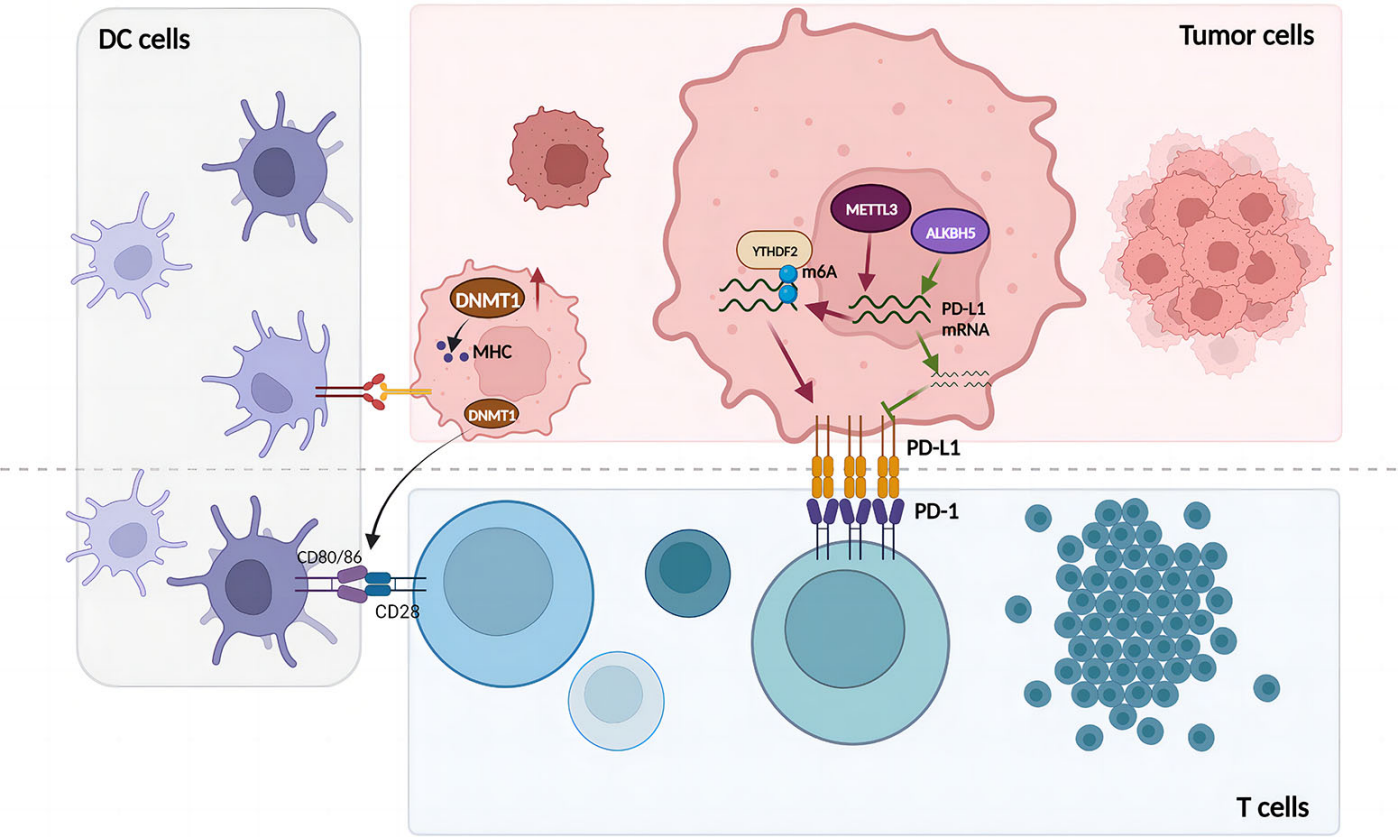
Roles of some N6‐methyladenosine (m6A) and N7‐methylguanosine (m7G) regulatory enzymes in tumour immunity. PD1 is a programmed death receptor expressed on T cells. A programmed death ligand (PD‐L1) is expressed in tumour cells. The combination of PD1/PD‐L1 will make T cells lose their killing ability. CD80 and CD86 bind to the receptor CD28 protein on the surface of T cells, give the signal to the activation of naive T cells, produce a stimulating effect and promote the activation, proliferation and differentiation of T cells.

In recent years, the impact of m7G‐related regulators on tumour immunity has received increasing attention. For example, NCBP2 and EIF4E3 can influence the immune environment and response to immunotherapy, thereby inducing tumour transformation in patients with HNSCC.^130^ Another study showed that clinical adrenocortical carcinoma (ACC) patients with high METTL1 expression had lower levels of CD8+ T‐cell infiltration and higher levels of macrophage infiltration compared with those with low expression, suggesting that METTL1 exerts a profound effect on tumour immunity in ACC^131^ (Figure 3).

## 4 RNA METHYLATION‐RELATED INHIBITORS AS THERAPEUTIC TARGETS

**FIGURE 4:**
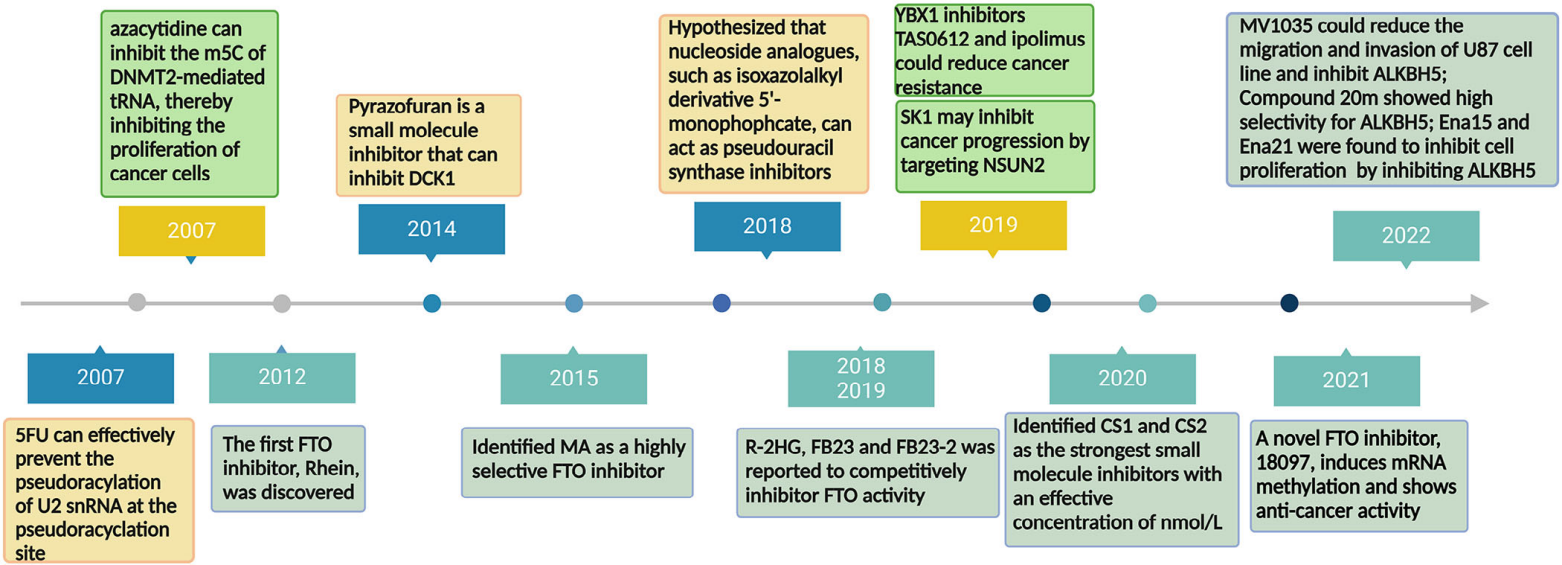
Timeline of discovery of specific inhibitors of partial regulators of N6‐methyladenosine (m6A), 5‐methylcytosine (m5C) and pseudouridine modification is presented.

In recent years, as research into RNA modification has deepened, the research on the inhibitors of related modification enzymes has also developed rapidly. From 2012 to 2021, research on FTO inhibitors has evolved rapidly; 2012 saw the discovery of rhein, the first FTO inhibitor; 2015 saw the discovery of MA as a more potent FTO inhibitor; 2018−2019 saw the proposal that R‐2HG, FB23 and FB23‐2 could competitively inhibit FTO activity; 2020 saw the discovery of CS1 and CS2, two more powerful inhibitors; 2021, a novel FTO inhibitor, 18097, is found to induce mRNA methylation and show anticancer activity. Meanwhile, studies on ALKBH5 inhibitor, another m6A demethylase, are also gradually emerging. Two effective inhibitors MV1035 and compound 20m were found in 2022. There are relatively few studies on inhibitors of m5C‐modifying enzymes. In 2007, a study found that m5C methylase DNMT2 mediated modification of m5C could be completely inhibited by azacytidine, thereby reducing the proliferation of cancer cells. YBX1 inhibitors, TAS0612 and ipolimus, and NSUN2 inhibitor SK1 were discovered in 2019. Research on inhibitors of pseudouridine modifying enzymes is also progressing gradually. In 2007, a pseudouridine inhibitor, 5fu, was found to inhibit pseudouridine synthase through a uracil‐like configuration in RNA. Pyrazofuran was found to be a DCK1 inhibitor in 2014. In 2018, a study proposed that nucleoside analogues, such as isoxazolyl‐derived 5′‐monophosphates, may act as competitive inhibitors of pseudouridine 5′‐monophosphate glycosylase (Figure 4).

### 4.1 m6A inhibitors and their roles in cancer

The complex interplay between m6A demethylases and RNA metabolism has been revealed by epigenetic studies in recent decades.^132^, ^133^ Among them, many recent studies have focused on two demethylases, FTO and ALKBH5. Numerous research advances have many research breakthroughs that have underscored the oncogenic significance of FTO and ALKBH5 across a spectrum of cancers. There is growing evidence that FTO and ALKBH5 are promising new treatment targets for several human disorders, particularly tumours. The identification of small molecule inhibitors opens up new avenues for therapeutic intervention. This section provides a brief summary of recent progress in the development of inhibitors specifically targeting FTO and ALKBH5. Furthermore, a comprehensive review is conducted on diverse screening methodologies grounded in various principles.

#### 4.1.1 FTO inhibitors

##### Rhein acid

Chen and coworkers discovered rhein, a natural product, in 2012, marking the advent of the first FTO inhibitor. It is neither a 2OG analogue nor an iron chelator, but a competitive substrate inhibitor of FTO.^134^ Research has the synergistic effect of rhein in conjunction with TKI that offers a comprehensive strategy for eradicating TKI‐resistant leukaemia cells.^135^

##### Meclofenac

In 2015, meclofenac (MA) was identified by Huang and coworkers as a new inhibitor of FTO.^136^ Similarly, MA, devoid of any chemical resemblance to 2OG or iron complexation, is anticipated to competitively bind with FTO. Furthermore, this study illustrates that MA has the ability to impede the suppression of FTO's m6A demethylase activity.

##### FB23 and FB23‐2

FB23 and FB23‐2 were FTO inhibitors developed by structural optimisation and design. Both inhibit FTO by binding to FTO and specifically inhibiting m6alpha demethylase activity. Compared with FB23, FB23‐2 has better cell permeability, activity and selectivity.^137^

##### CS1 and CS2

CS1 and CS2 have been identified as the most potent small molecule inhibitors of FTO.^138^ They have shown strong anti‐leukaemic effects in a mouse model. Moreover, they have exhibited inhibitory effects on the growth of glioblastoma, breast and pancreatic cancer cells, alongside displaying significant efficacy against BRCA in mouse models.

##### R‐2HG

In 2018, the endogenous tumour metabolite R‐2HG was reported to competitively inhibit FTO enzyme activity. This study demonstrated that R‐2HG has multiple anti‐leukaemic effects, reducing the viability of leukaemia cells while also exacerbating the progression of leukaemia cell death. Additionally, R‐2HG has demonstrated antitumour efficacy in glioma.^16^

##### 18097

**FIGURE 5:**
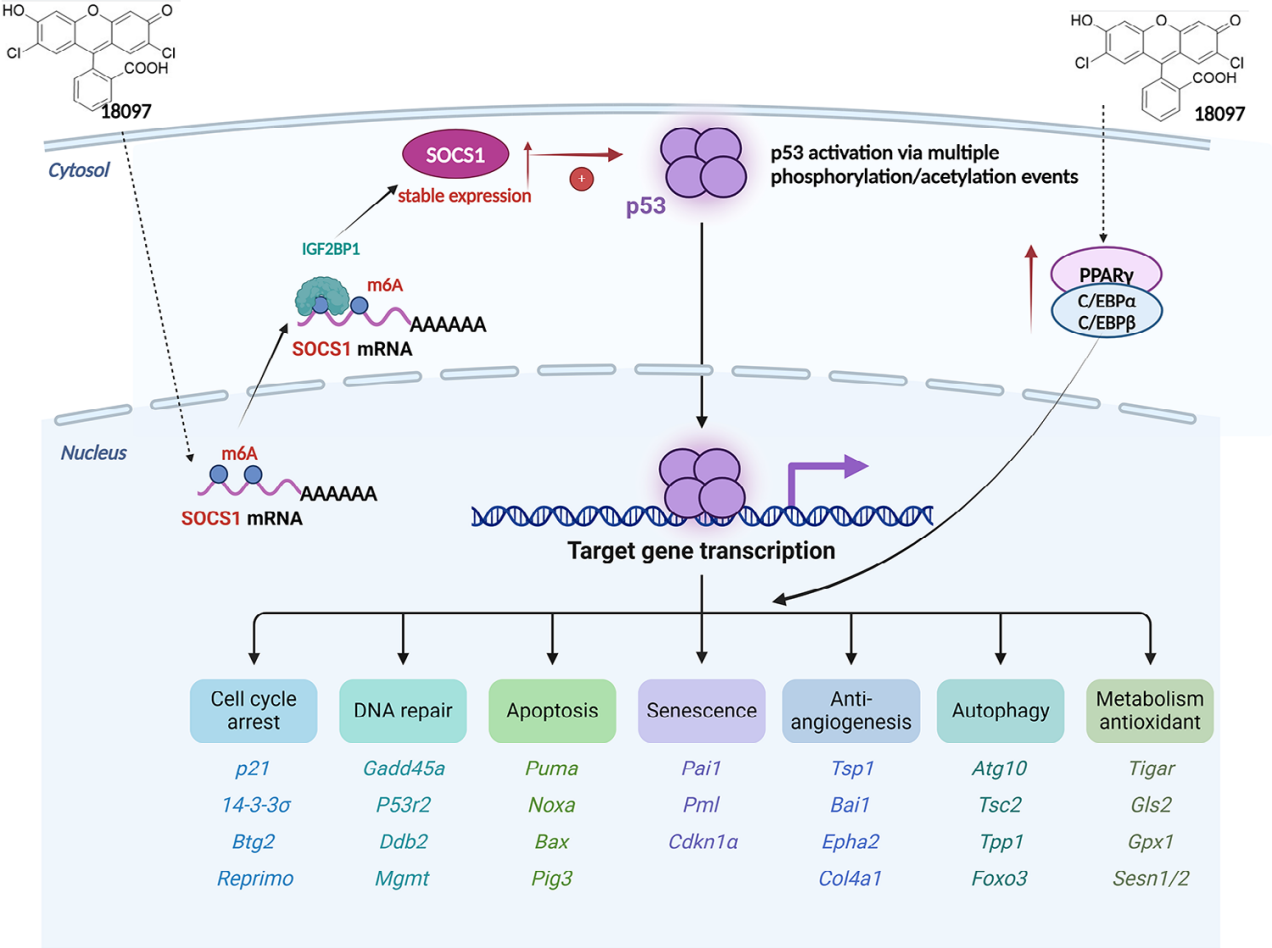
Molecular mechanism of action of the FTO inhibitor 18097. As a potent inhibitor of FTO, 18097 was able to increase the N6‐methyladenosine (m6A)‐modified abundance of cytokine signalling pathway suppressor 1 (SOCS1), and furthermore to increase the stability of SOCS1 by recruiting IGF2BP1 and subsequently activating the P53 pathway. In addition, 18097 inhibited cellular adipogenesis by downregulating peroxisome proliferator‐activated receptor γ (PPARγ), CCAAT/enhancer‐binding protein α (C/EBPα) and C/EBPβ.

In 2021, researchers investigated a new inhibitor of m6A demethylase FTO, called 18097, which selectively inhibits FTO demethylase activity, induces mRNA methylation and exhibits more potent anticancer activity^139^ (Figure 5).

### 4.2 ALKBH5 inhibitors

ALKBH5 has a key role in many important cellular procedures and contributes to the development and progression of cancer. Hence, ALKBH5 is recognised as a promising target for anticancer therapeutics, garnering significant interest in the search for its inhibitors in recent years. However, up to now, only a few ALKBH5 inhibitors have been documented. One such example is imidazole‐benzoxazine‐5‐thioone (MV1035), a novel sodium channel blocker, which has undergone testing in tumour cells of various origins. Nevertheless, Nicolini and coworkers primarily notable decrease the impact of MV1035 on mitigating the metastasis of the U87 cell, along with a suppression of ALKBH5.^140^ In addition, Simona Selberg et al. showed that 2‐[(1‐2‐hydroxy‐2‐oxygen radicals‐phenylethyl) sulphonyl] acetate and 4‐[(furan‐2‐) methyl] amino‐1,2‐diazine alkanes‐3,6‐dione underwent validation through high‐throughput virtual screening, complemented by an m6A antibody‐based ELISA. It was also validated in the cell proliferation of leukaemia cell lines.^141^ Reports of effective and specific ALKBH5 inhibitors remain scarce despite these recent advances. Therefore, several new ALKBH5 inhibitors have been investigated. Among these, 20m, a potent ALKBH5 inhibitor, was shown to exhibit remarkable selectivity for both ALKBH5 and FTO. In the CETSA assay, the expression of ALKBH5 was effectively stabilised in HepG2 cells for 20 min. In subsequent in vitro cell experiments, it notably hindered m6A demethylation within intact cells. These findings underscore 20m's potency, selectivity and cell‐permeable properties, positioning it as a valuable tool for probing the biological functions of ALKBH5.^142^ In addition, two novel anti‐AlkB inhibitors, Ena15 and Ena21, were identified. These two inhibitors, by blocking the cell cycle, increasing m6A levels and stabilising FOXM1 mRNA expression, effectively suppressed cell growth in glioblastoma multiforme cells.^143^

### 4.3 m5C inhibitors

To date, relatively few specific m5C‐related modifying enzyme inhibitors have been developed. As mentioned above, m5C, in conjunction with its regulators such as NSUN2, plays a significant role in the initiation and growth of various tumour; hence, it is a potential therapeutic target for disease intervention.^30^ Meanwhile, NSUN2 expression was diminished upon inhibition of sphingosine kinase (SPHK), a key player in preserving the equilibrium of sphingolipid metabolism throughout cellular proliferation.^144^ Consequently, the SPHK1 inhibitor SK1 could be a candidate anticancer treatment by targeting NSUN2.^145^ Furthermore, recent research indicates that TAS0612 and ipomus targeting the m5C ‘reader’ YBX1 have been demonstrated to diminish drug resistance in cancer therapy.^146^ However, whether YBX1 inhibitors act in an m5C‐dependent manner is unknown and requires further investigation. In addition, in a study by Lyko and coworkers, it was found that DMNT2‐mediated m5C could be completely inhibited by azacitidine in cancer cells, resulting in decreased cancer cell proliferation.^147^ Hence, directing focus towards DNMT2 presents itself as a promising avenue for the development of potent epigenetic drugs in cancer therapy.

### 4.4 Pseudouridine inhibitors

**FIGURE 6:**
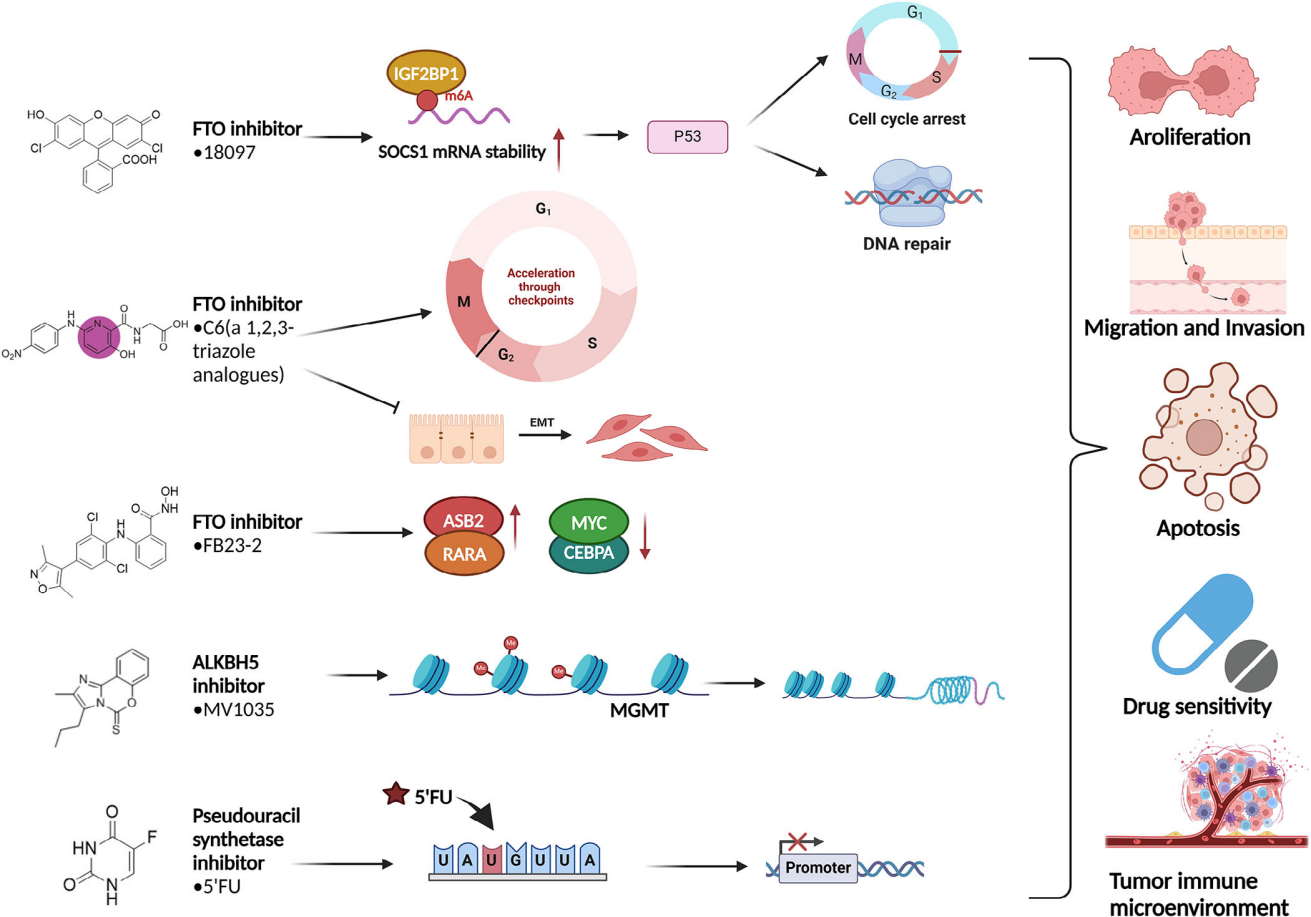
FTO, ALKBH5 and pseudouridine synthase partial inhibitors and their inhibitory pathways are introduced. O6‐methylguanine‐DNA‐methyltransferase (MGMT) is a DNA repair protein. ASB2 is an ankyrin repeat containing SOCS box 2, and retinoic acid receptor alpha (RARA) is a retinoic acid receptor, both of which are related to protein stability. CEBPA is the transcription factor CCAAT enhancer‐binding protein α, and MYC is expressed by a class of nuclear protein oncogenes. Both are related to differentiation and proliferation. These inhibitors inhibit cancer progression by regulating related pathways.

From a clinical perspective, PUSs or Ψ may be potential anticancer targets and biomarkers. However, although alterations in DCK1 expression have been extensively documented in cancer, there remains a lack of understanding regarding the status of other Ψ synthetases. Currently, several small molecule inhibitors targeting PUSs are trying to inhibit cancer progression by reducing the activity of DCK1, but the effect is not obvious.^148^, ^149^, ^150^ Pyrazofuran, a compound known for inhibiting otodine‐5′‐monophosphate decarboxylase (ODCase), also demonstrates inhibition of DKC1. However, whether pyrazofuran has an effective therapeutic effect in patients with DKC1 overexpression is unknown. Another promising small molecule inhibitor is 5‐Fu, which has demonstrated clinical effectiveness in enhancing survival rates among patients battling various types of cancer.^151^ It has been shown that 5‐Fu can inhibit pseudouridine synthetase through the configuration of a uracil analogue in RNA.^152^ Moreover, in a separate investigation, Floresta et al. suggested that nucleoside analogues might function as inhibitors of pseudouridine 5′‐monophosphate glycosidases, thereby competing with the natural substrates.^149^ In summary, these studies provide a basis for continuing to search for potential Ψ‐synthase inhibitors and using them to treat cancer (Figure 6).

## 5 DISCUSSION

RNA modifications have an impact not only on normal physiological activities but also on pathological conditions such as inflammation, infertility, neurological disorders and cancer. The advent of high‐throughput next‐generation sequencing (NGS) and other sequencing methodologies has significantly enhanced our comprehension of the overall extent of RNA methylation. Moreover, at present, N6‐methyladenine detection technology is also gradually improving. For example, for the highly sensitive and site‐specific detection of m6A, a novel electrochemical biosensor was developed using double hindered replication and nucleic acid‐coated MB @Zr‐MOF.^153^ With the increased understanding of RNA methylation, aberrant expression of the relevant modifying enzymes in tumour tissues is frequently found, and this dysregulation severely affects the prognosis of cancer patients. Currently, the academic community is understanding how such RNA modification abnormalities affect the progression of human diseases, including cancer. What is more novel is that in recent years, many emerging studies have reported environmental factors in RNA modification. For example, through nitrite induction, it has been found that m6A levels increase and further activate AKT1 and transmit proliferation signals to ribosomal biogenesis, which is a major contributor to heavy metal carcinogenesis.^154^ Other studies have found that MNNG, as a typical environmental chemical carcinogen, is strongly associated with the occurrence of GC, and that prolonged exposure to MNNG increases the expression of METTL3 and EMT markers.^155^ Similarly, triclosan (TCS), a typical anthropogenic pollutant, can reduce m6A modification by targeted upregulation of ‘erasers’ and downregulation of YTHDF1, leading to disruption of lipid metabolism.^156^ Recent research advances in the field of epigenomics (epigenetic genomics) have suggested a link between reprogramming of elements involved in the epigenetic transcription machinery and the development of cancer. The dynamic expression patterns of these regulators make it difficult to determine the specific consequences of aberrant accumulation of modifications in RNA metabolism. In this review, we provide a comprehensive overview of the distributional characteristics, regulatory factors and potential functions of various types of RNA methylation in different RNAs.

The identification and functional investigation of regulatory factors involved in RNA methylation have significantly advanced our comprehension of this process. Moreover, RNA methylation exerts a crucial influence on the regulation of gene transcription, expression, stability and degradation, thereby manifesting a multifaceted impact on cancer. This article describes the different functions of methylation regulators in different types of cancer. The article highlights how RNA modifications can reduce the stability of oncogenes and promote tumour progression. Conversely, these modifications can also play an inhibitory role in cancer progression. This review provides an overview of the functions of specific methylation regulators in various cancers.

A comprehensive understanding of RNA methylation has revealed the potential reversibility of many RNA methylation modifications and epitope transcriptional abnormalities, offering great promise for epitope transcription therapy. In recent years, a number of inhibitors targeting RNA‐modified proteins have been identified. For example, some cytidine analogues, which inhibit cytosolic m5C methylase, have gained clinical approval for the treatment of haematological malignancies.^157^ The majority of research on inhibitors primarily focuses on m6A‐modified proteins, as extensively discussed in this review. In addition, it is noteworthy that nanomedicine based technologies have been used to explore RNA drug therapies. For example, a biologically coupled nanoparticle called HA‐P5 was generated by coupling an FGF2‐derived endogenous peptide (P5) to HA polysaccharide. This nanoparticle inhibits downstream molecules of YTHDF3 in SZ95 cells.^158^ However, several unresolved issues persist. First, our current knowledge does not allow for the precise determination of the spatiotemporal modification of the transcriptome in question. Additionally, the development and utilisation of therapeutic agents remain inadequately addressed, as there is insufficient consistency in verifying the carcinogenic or cancer‐inhibitory effects resulting from aberrant deposition of these RNA modifications. Finally, there exists a considerable body of research on inhibitors associated with m6A modification, whereas the exploration of other types of modifications remains limited.

Given the association between RNA methylation and various cancer types, our research findings possess the potential to significantly influence cancer therapy through the targeting of epitranscriptomic RNA methylation. It is important to note that the clinical implementation of compounds aimed at RNA methylation is currently in its infancy. In subsequent investigations, there is a possibility to enhance the efficacy of existing inhibitors by modifying and optimising their structural basis. Consequently, due to the challenges mentioned above, there is still a significant need for progress in the understanding of epigenetic modifications and the development of epigenetic inhibitors.

## AUTHOR CONTRIBUTIONS

Huanxiang Chen and Hongyang Liu drew the drawings and wrote the manuscript. Chenxing Zhang, Huanxiang Chen and Yang Li collected relevant literature. Junhu Wan, Qiaozhen Kang and Huihui Gu provided guidance and guidance throughout the preparation process. Ruike Zhang and Xiangzhuan Zhao contributed to the revision of the manuscript. All authors read and approved the final manuscript.

## CONFLICT OF INTEREST STATEMENT

The authors declare they have no conflicts of interest.

## ETHICS STATEMENT

Not applicable.

## CONSENT FOR PUBLICATION

Not applicable.

## Data Availability

Not applicable.
